# Artificial intelligence and synthetic biology in traditional Chinese medicine: revolutionizing public health applications

**DOI:** 10.3389/fpls.2026.1789960

**Published:** 2026-04-13

**Authors:** Shanshan Han, Tao Qin, Zhizhen Feng

**Affiliations:** 1 Shaanxi Key Laboratory of Qinling Ecological Security, Enzyme Engineering Research Center of Shaanxi Province, Xi’an, China; 2 Bio-Agriculture Institute of Shaanxi Province, Shaanxi Academy of Sciences, Xi’an, China

**Keywords:** artificial intelligence, precision medicine, sustainable development, synthetic biology, traditional Chinese medicine

## Abstract

Traditional Chinese Medicine (TCM) has played a vital role in public health throughout history, particularly evidenced during the COVID-19 pandemic, where it demonstrated both accessibility and clinical efficacy. However, TCM faces critical challenges, including unsustainable medicinal resources, ambiguous multi-target mechanisms, and a lack of standardized clinical evaluation systems. Addressing these issues requires interdisciplinary integration, particularly between synthetic biology and artificial intelligence (AI). Synthetic biology offers solutions to resource scarcity and production standardization by enabling the sustainable biosynthesis of active compounds. Meanwhile, AI enhances TCM research through bioinformatics-driven compound prediction, machine learning-assisted quality control, and network pharmacology-based mechanism elucidation. AI also improves diagnostic reproducibility, aligning with synthetic biology’s precision-driven framework. Together, these technologies facilitate the transformation of TCM from an experience-based practice into a standardized, evidence-based public health intervention. This review highlights the synergistic potential of AI and synthetic biology in overcoming TCM’s modernization barriers. By leveraging AI for data-driven drug discovery and synthetic biology for scalable production, TCM can achieve sustainable development while retaining its therapeutic value. Future efforts should focus on enhancing AI interpretability, expanding biological databases, and optimizing cross-disciplinary collaboration to fully realize this integration.

## 1 Introduction

### 1.1 Modern challenges of traditional Chinese medicine in public health

Traditional Chinese Medicine (TCM) has made significant historical contributions to public health. Special treatises such as *Treatises on the Pestilence* have established a complete system that combines the innovation of disease differentiation prescriptions with prevention and control (Zheng et al., 2023). During the COVID-19 pandemic, TCM also revalidated its accessibility advantage and high application value (Huang et al., 2023). However, TCM is facing a crisis of resource sustainability, with ambiguous multi-target mechanisms, making it difficult to establish a standardized clinical evaluation system (Wang et al., 2021; Zhang et al., 2023). Although single herbal medicines have demonstrated clear efficacy, the broader system of TCM has historically lacked molecular-level scientific validation (Wang et al., 2021; Gan et al., 2023).

Facing contemporary public health demands, the conservation of medicinal resources, deconvolution of complex systems, standardization of production, and technological innovation constitute critical bottlenecks. To overcome these challenges, deep integration of synthetic biology with artificial intelligence and cross-disciplinary collaboration is imperative (Lin et al., 2022; He et al., 2025; Wang et al., 2025).

### 1.2 The inevitability of technological integration

Artificial Intelligence (AI) addresses complex data analysis. Through bioinformatics and machine learning, targeted TCM compounds can be predicted (Cao et al., 2024). Machine learning provides convenience for clinical diagnosis and quality control of TCM (Chen and He, 2022). Network pharmacology (NP) offers a novel approach to elucidate and visualize the complex interaction networks of TCMs in treating multifactorial diseases (Li et al., 2023). Current AI models rely on database integrity and have insufficient interpretability. It is necessary to combine different technologies to enhance reliability (Zhang et al., 2024).

Synthetic biology addresses resources and standardization challenges, while machine learning elucidates prescription compatibility patterns to guide the reconstruction of bioactive combinations (Wang et al., 2021).

### 1.3 How this review catalyzes interdisciplinary integration

AI plays a pivotal role in advancing the sustainability of TCMs by guiding synthetic biology approaches. Through AI-powered screening of active components from scarce medicinal resources, synthetic biology can be directed toward constructing cell factories for sustainable bioproduction.

Regarding the sustainability of TCMs, AI provides critical guidance for synthetic biology. By leveraging AI to screen active components from scarce medicinal resources, synthetic biology can be directed toward constructing cell factories for sustainable bioproduction.

**Figure 1:**
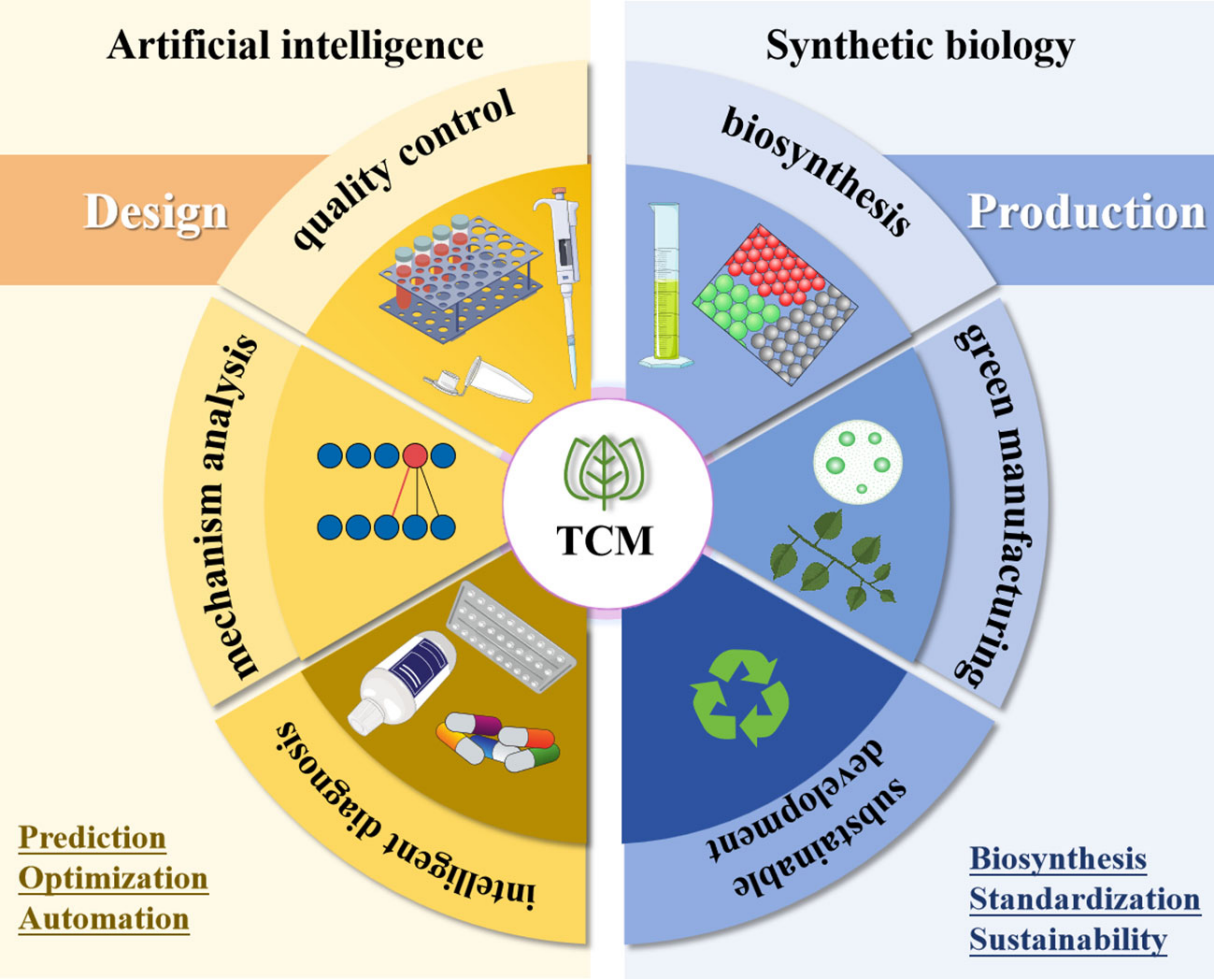
Schematic diagram.

Furthermore, AI facilitates the quantification and standardization of TCM diagnosis (Wang et al., 2021), rendering it a reproducible methodology. This paradigm aligns intrinsically with the standardized production framework of synthetic biology. Collectively, these synergies transform TCM from an individualized experiential practice into a standardized public health intervention tool (Zhang et al., 2023)(**Figure 1**).

## 2 The crucial applications of AI in TCMs

### 2.1 Quality control

**Figure 2:**
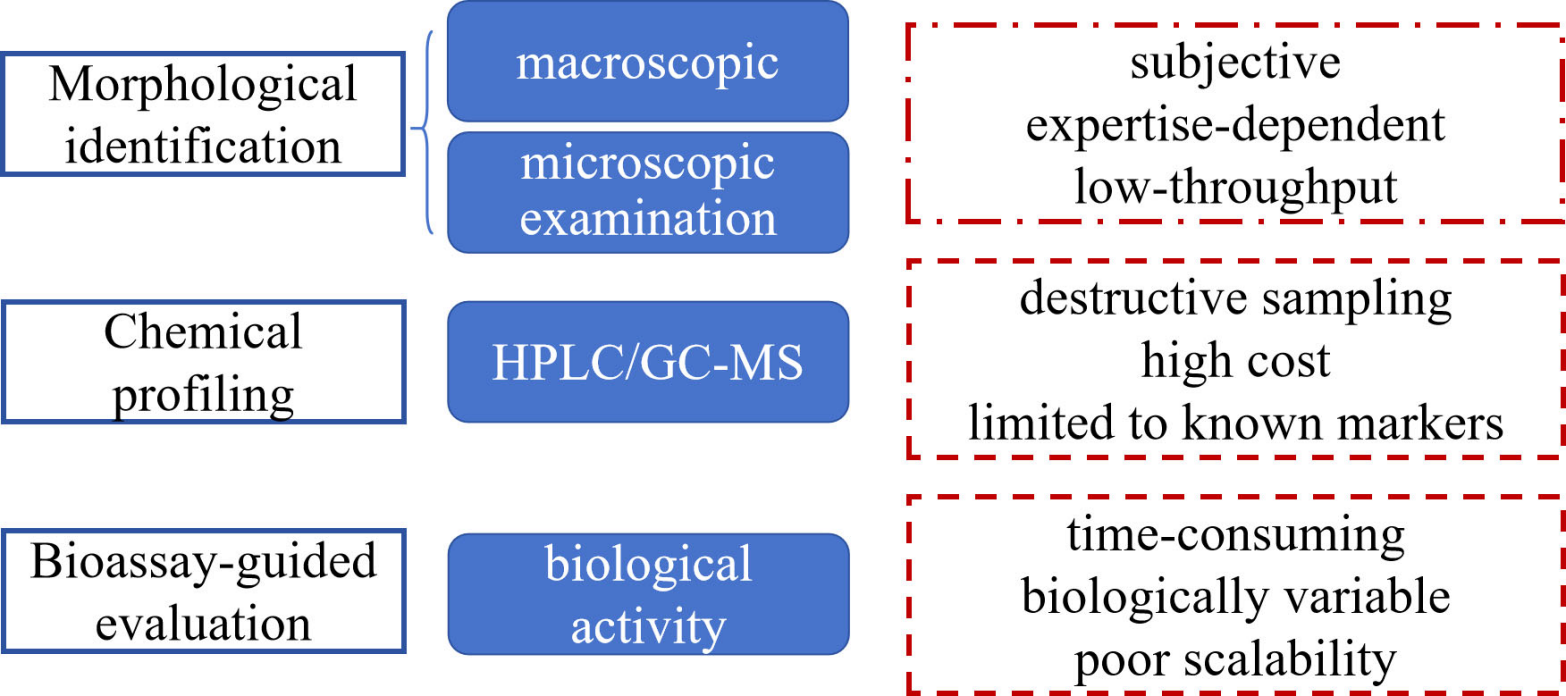
Traditional TCM QC approach.

Traditional quality control (QC) of TCMs primarily relies on three pillars: morphological identification, chemical profiling and bioassay-guided evaluation (Zhong et al., 2009; You et al., 2018; Zhang et al., 2022) (**Figure 2**). These conventional approaches, however, exhibit significant limitations in addressing: (1) batch-to-batch variability caused by environmental factors, (2) pervasive adulteration and counterfeiting risks due to subjective assessment methods, and (3) dynamic processing effects unmonitored by endpoint assays (Wu et al., 2018; Ming-Liang et al., 2022; Cai et al., 2023; Li et al., 2025). For instance, the polysaccharide content in *Astragalus membranaceus* can vary greatly due to different growth regions, making it very easy to be adulterated (Guo et al., 2023).

Compare with conventional QC methods, AI transforms multisource data into quantifiable QC parameters (Lv et al., 2023b), enabling an integrated framework through four technological pathways (**Table 1**):

**Table 1:** Performance comparison of AI vs. conventional QC methods.

QC parameter	Conventional method	AI method	Advantages of AI method	Reference
Active ingredients quantification	HPLC	Raman spectroscopy + CNN	No sample destruction High Accuracy High Sensitivity High throughput	(Yan et al., 2020)
Adulteration detection	DNA barcoding	FTIR + Random Forest		(Wang et al., 2018; Zhao et al., 2020)
Species authentication	Microscopy	ResNet-152 image analysis		(Jiang et al., 2021)
Geographic origin tracing	Stable isotope analysis	Hyperspectral imaging + ML		(He et al., 2017; Ping et al., 2024; Wu et al., 2025)
Toxicity prediction	Post-processing HPLC	NIR + LSTM		(Zulkifli et al., 2023; Bai et al., 2024; Chung et al., 2024)

1. Sensory intelligence replacing subjective evaluation, demonstrated by computer vision systems (e.g., IRIS VA400 E-eye achieving 91.38% classification accuracy for *Cornus fructus (*YongXia et al., 2019)) and cost-efficient electronic nose applications for *Dendrobium* species discrimination (Wang et al., 2019);
2. AI-decoded chemical fingerprinting identifying activity-based markers via network pharmacology and enabling non-destructive quality assessment through hyperspectral imaging combined with machine learning (Guo et al., 2015; Zhang et al., 2022; Pan et al., 2024);
3. Multimodal data fusion facilitating real-time contaminant monitoring such as heavy metal detection (Singh et al., 2023; Bae et al., 2024);
4. Blockchain-enabled traceability, collectively constituting an integrated approach to enhance quality assessment.

Collectively, this framework establishes automated, objective QC with multi-scale verification capabilities from molecular profiling to macroscopic characterization.

### 2.2 Active ingredient mining and mechanism analysis

Conventional approaches for identifying bioactive compounds in TCMs, with low efficiency isolation, poor target prediction and synergistic ignorance, is facing significant challenges (Qiu et al., 2015; Wang et al., 2016; Haider et al., 2025). AI revolutionizes this paradigm through multi-omics data integration and predictive modeling (Pan et al., 2024).

For example, the TCMBank database (contains 61,966 ingredients, 15,179 targets) enables systematic elucidation of herbal compounds and discovery of novel therapeutic candidates (Lv et al., 2023a; Ge et al., 2024); Low-cost computational platforms, such as a credit card-sized computer retailing for <50 USD, can be used to predict biosynthetic pathways for terpenoids in *Artemisia annua (*Tan and Mutwil, 2020).

AI have deep learning-based virtual screening, is useful for structure activity prediction and optimization. DeepDTA and Co-VAE could identify drug-target (DT) interactions (Öztürk et al., 2018; Li et al., 2022), both are state-of-the-art method for DT binding affinity prediction, while ADMETlab 2.0 is important in the evaluation of excretion and toxicity of TCM components (Xiong et al., 2021).

AI could explain mechanism elucidation of TCM from compounds to pathways. DeepTCM has gone through multilevel model calibration and validation against a comprehensive set of herb and disease data so that it accurately captures the complex cellular signaling, molecular and theoretical levels of TCM (Qian et al., 2024). Meta-analysis using Bayesian networks could analyze the composition of TCM preparations, conduct association rule mining, and identify key herbs (Weng et al., 2023; Duan et al., 2024).

AI-driven discovery of bioactive compounds and mechanism elucidation in TCMs, enabling resource-efficient innovation, precision compound discovery, and multi-scale mechanism mapping (Zhao et al., 2024; Wang et al., 2025; Yang et al., 2025).

### 2.3 Intelligent diagnosis and formula personalization

TCM diagnosis traditionally relies on the Four Diagnostic Methods, which are inherently subjective and non-quantifiable (Li et al., 2022). For instance, the complexity of indicators in tongue diagnosis results in accuracy that varies by practitioner’s experience (Lo et al., 2015; Gan et al., 2025), while pulse diagnosis symptom patterns are not supported by standardized metrics (Wu et al., 2017; Zhu et al., 2023; Tang et al., 2024). AI addresses these challenges through the integration of multimodal data and predictive modeling (Li et al., 2022).

Objective quantification of diagnostic parameters. A convolutional neural network (CNN) based tongue image recognition system was developed using deep learning and visual question answering for interactive health detection (Zhuang et al., 2022). Maps symptom-herb-syndrome relationships in clinical records through deep learning (Sun et al., 2022).

AI offers heuristic guidance for the rationalization and optimization of TCM formulations (Cui et al., 2017; Chung et al., 2024; Galkin et al., 2025). By transforming TCM from an artisanal practice to a data-driven precision medicine, AI is fundamentally restructuring the field, replacing subjective, experience-dependent paradigms with quantifiable, evidence-based frameworks (Zhang et al., 2023; Long et al., 2025; Yang et al., 2025).

### 2.4 Assisting the construction of synthetic biology system for active ingredients of TCM

Synthetic biology designs and constructs new biological components, devices and systems through engineering principles, or redesigns existing natural biological systems to achieve specific functions (Chao et al., 2015).

In the field of TCM, synthetic biology has broad application prospects. Using synthetic biology technology, people can synthesize the target active ingredients of traditional Chinese medicine by introducing enzymes related to the synthesis of active ingredients or precursors of active ingredients into other biological platforms (such as tobacco, Escherichia coli, yeast, etc.), or improve the expression of the target active ingredients in traditional Chinese medicine by genetic modification.

Synthetic biology achieves efficient and sustainable production of active compounds in TCM, providing a new approach for the production of active compounds in TCM development. This addresses the resource shortages and environmental pressures faced by TCM materials, while also realizing standardized production of active compounds in TCM, improving product quality and stability (Feng et al., 2022; Guo et al., 2024).

Due to the complexity of living systems and the lack of rational design principles, repeated trial and error experiments are often necessary. Traditional synthetic biology system construction relies on time-consuming and labor-intensive manual design, labor-intensive testing, and detailed analysis, which is inefficient. The introduction of AI enables people to quickly process massive amounts of biological data, thereby significantly improving the efficiency and accuracy of this process (Tang et al., 2020; Dhivya et al., 2021; Guo et al., 2023; Gong et al., 2024).

On the one hand, AI can realize the annotation of huge genome and protein family and the functional prediction of a variety of enzymes through deep learning, high-throughput analysis and other methods. On the other hand, AI can realize the optimization and even *de novo* design of proteins with specific functions through calculation, simulation and other methods. At a higher level, AI can assist in the design of biosynthetic pathways for synthetic biology systems. All these indicate the great potential of AI for TCM biosynthesis (Gong et al., 2024).

## 3 Synthetic biology drives the development of TCM

### 3.1 Lack of resources restricts the development of TCM

With the deepening of research on TCM, TCM has gradually shifted from the plant level to the molecular level. Modern practitioners of TCM began to study the role of the active ingredients in traditional Chinese medicine on diseases and extracted the active ingredients from plants to make drugs for clinical treatment.

Up to now, a series of drugs based on the active ingredients of traditional Chinese medicine, such as gastrodin and vincristine, have been put into clinical practice (Luan et al., 2023).

However, the content of many active ingredients in plants is very low, which greatly limits their transformation into patented drugs for clinical use or has been converted to patented drugs but the yield is too low to be used on a large scale (Dai and Wang, 2023).

Take ginsenoside Rh_2_, a common TCM active compound, as an example. It has high anti-tumor activity and no adverse reactions to normal cells. However, in normal ginseng medicinal materials, the content of ginsenoside Rh_2_ is extremely low (1.0–3.0 μg/g DW) (Hu et al., 2020). In addition, the production of ginseng, its main source, is also very limited. During the development of TCM, people have attempted to provide more ginseng for the clinical practice of TCM by growing ginseng. However, as a precious medicinal plant, ginseng is confronted with serious obstacles to continuous cropping. Moreover, the cultivation of ginseng can also cause a series of negative impacts on the environment, such as soil acidification, accumulation of toxic compounds, and a decrease in bacterial diversity (Dong et al., 2018).Therefore, it is almost impossible to use it alone for clinical anti-tumor treatment, because the yield from ginseng is too low and the cost is too high.

Cases like ginsenoside Rh_2_ are by no means isolated. Many active compounds of TCM materials, such as triptolide derived from Tripterygium wilfordii, gastrodin derived from Gastrodia elata and artemisinin derived from Artemisia annua, etc.,face the problem of high clinical demand and limited plant source production (Heijden et al., 2004; Luo et al., 2007; Liu et al., 2011; Zeng et al., 2013; Tang et al., 2018).

Fortunately, with the development of synthetic biology, the heterologous biomimetic synthesis of active compounds in traditional Chinese medicine that has emerged in recent years is expected to solve the problem of the mismatch between the insufficient resources of TCM and the excessive clinical demand (Dai and Wang, 2023).

### 3.2 Promising synthetic biology in TCM

To date, significant progress has been made in the heterologous biomimetic synthesis of active compounds derived from traditional Chinese medicine, leveraging the principles of synthetic biology:

#### 3.2.1 Biosynthesis of ginsenoside Rh_2_

Based on the already discovered synthetic pathway of ginsenoside Rh_2_ in ginseng, Qin et al. screened out the genes related to the synthesis of ginsenoside Rh_2_ (*PnDDS, CYP12H* and *UGTPn3*) from Panax notoginseng, a traditional Chinese medicine plant of the same family and genus as ginseng, and introduced these genes into tobacco, successfully achieving the heterocyclic synthesis of ginsenoside Rh_2_ in tobacco. Moreover, the content of ginsenoside Rh_2_ in this transgenic tobacco plant can reach 2.00-5.30μg/g DW, which is significantly higher than the average ginsenoside content in ginseng plants (Chen et al., 2023).

#### 3.2.2 Biosynthesis of triptolide precursor

Triptolide is an active diterpenoids extracted from Tripterygium wilfordii, a traditional Chinese medicinal herb, which are resistant to inflammation, immunosuppression, renal fibrosis and anti-tumor. At present, triptolide is extracted from the Tripterygium wilfordii and is separated from its compound. However, due to the low amount of the triptolide in Tripterygium wilfordii, the current production is still not enough to meet the clinical needs. Therefore, people begin to study the synthesis of triptolide in other biological or chemical methods (Gao et al., 2021).

Hu et al. constructed a chimeric miltiradiene synthase by combining the class II diterpene synthase (di-TPS) CfTPS1 from Coleus *forskohlii* (Plectranthus *barbatus*) and the class I di-TPS SmKSL1 from Salvia miltiorrhiza. This chimeric enzyme was expressed in yeast to catalyze the conversion of (E,E,E)-geranylgeranyl diphosphate (GGPP) into miltiradiene, an essential precursor for triptolide biosynthesis. This technology achieved efficient conversion of GGPP to miltiradiene (550.7 mg L^-1^ in shake flasks and 3.5 g L^-1^ in a 5-L bioreactor) (Hu et al., 2020).

This technology provides an efficient and green process for the production of the important intermediate and lays an important foundation for the further biosynthesis of triptolide,making it possible to further expand the clinical application of triptolide in the future.

#### 3.2.3 Biosynthesis of gastrodin

Gastrodin is mainly extracted from the traditional Chinese medicine Tianma, which is the steamed and dried rhizome of G. elata Blume. Although G. elata Blume has been cultivated artificially for a long time, its price is very expensive due to the high time cost and labor cost. In addition, the content of gastrodin in Tianma was extremely low, with an average of only 0.41% (Tang and Eisenbrand, 1992).

Many efforts have been made to improve the production of gastrodin. In 2016, Bai et al. constructed a biosynthesis system of gastrodin from glucose in Escherichia coli by CAR from Nocardia, Sfp from Bacillus, endogenous ADHs and Rhodiola UGT73B6. Highly efficient biosynthesis of gastrodin (titer of about 545 mg L^−1^) was achieved (Bai et al., 2016).

#### 3.2.4 Biosynthesis of artemisinin

Artemisinin, an antimalarial drug extracted from the TCM Artemisia annua, has saved tens of millions of lives. However, the low concentration of artemisinin in artemisia annua, which represents only 0.01% to 0.8% of the dry weight of the plant, makes artemisinin relatively expensive to meet the demand for the more than 100 million courses of artemisinin combination therapies per year. Therefore, it is necessary for people to explore more production methods of artemisinin. In this process, synthetic biology offers more possibilities for the mass production of artemisinin (Liu et al., 2011).

In 2013, paddon constructed a yeast biosynthesis platform for artemisinic acid, an important artemisinin precursor, using codon-optimized ADS, CYP71AV1, CPR1, CYB5, ADH1, and ALDH1 genes of artemisia annua. Mass production of artemisinic acid (25g/L) was realized. Artemisinin was further synthesized by chemical synthesis based on the biosynthetic artemisinic acid *in vitro*. The industrial biochemical synthesis of artemisinin was achieved (Paddon et al., 2013).

In order to further simplify the steps, Khairul Ikram et al. optimized the previous artemisinic acid biosynthesis system and introduced it into *Physcomitrella patens* in 2017. After synthesizing artemisinic acid in this biological platform, it further directly catalyzes the conversion of artemisinic acid to artemisinin by UV and oxygen. Complete biosynthesis of artemisinin was achieved. A high initial production of 0.21 mg/g dry weight artemisinin was observed after only 3 days of cultivation (Khairul Ikram et al., 2017).

## 4 TCM combined with AI helps public health

### 4.1 The unique advantages of TCM in the field of public health

Traditional Chinese medicine (TCM) plays a unique role in dealing with emerging infectious diseases. Its holism and syndrome differentiation and treatment are helpful to regulate the body and enhance resistance at the macro level (Zhang et al., 2015).

One of the core advantages of traditional Chinese medicine is its emphasis on the “holistic concept”, which believes that the human body is an organic whole, and the occurrence and development of diseases is the result of the comprehensive action of many factors. Therefore, TCM treatment is not limited to a single symptom or disease, but to achieve the purpose of treatment by regulating the overall balance of the body. Different from the drug resistance and toxic side effects that may be caused by single-target targeted treatment in modern medicine, traditional Chinese medicine (TCM) can achieve a more comprehensive rehabilitation effect through the synergistic action of multi-components, multi-targets and multi-pathways (Yue et al., 2013; Li et al., 2023).

Another core advantage of TCM is “syndrome differentiation and treatment”, that is, individualized diagnosis and treatment based on individual differences, disease stages, and physical characteristics of patients. This personalized program makes traditional Chinese medicine more flexible and precise in chronic disease management (Yue et al., 2013; Lin et al., 2015).

### 4.2 Prospects of “TCM+AI” in the future

The thought of “preventive treatment for disease” in traditional Chinese medicine (TCM) emphasizes the prevention of the occurrence and development of diseases, which is reflected in the management of chronic diseases as early intervention, delaying disease progression and reducing recurrence. This concept is highly consistent with the needs of modern health management (Lin et al., 2015).

Hopefully, in the future, with the development of portable health detection equipment and the progress of AI models combined with TCM theory. AI is expected to implement the concept of “prevention of disease” to everyone. Based on the personalized health data collected by health detection equipment, “AI+TCM”can give personalized health advice, making contributions to public health.

## 5 Conclusion

The convergence of artificial intelligence (AI) and synthetic biology is fundamentally transforming traditional Chinese medicine (TCM) from an experience-based practice into a data-driven public health solution. This paradigm shift is evidenced by three groundbreaking advancements.

### 5.1 Precision-driven standardization

AI overcomes TCM’s historical limitations in quality control and diagnostic subjectivity. ResNet-152 achieves 99% accuracy, 99% precision and 99% recall in tongue diagnosis (Jiang et al., 2021). The development of Q-markers proposed an integration model for TCM quality management (Bai et al., 2018).

### 5.2 Sustainable bioproduction

Synthetic biology enables scalable, eco-friendly manufacturing of rare TCM compounds (Wang et al., 2021; Zhong et al., 2022; Li et al., 2023). With yeast as the cell factory, the production of artemisinin was transferred from plant to yeast, increasing production to 25 g/L (Zhao et al., 2022).

### 5.3 Democratized healthcare access

Portable AI tools bridge urban-rural health disparities (Li et al., 2023). DeepTCM had conducted multi-level model calibration and validation for TCM and disease data, making AI-prescribed herbal formulas come true (Qian et al., 2024).

In order to fully realize these potential technique fusion, regulatory harmonization and global data commons need to be given special attention (Dubey et al., 2025). The collaboration between AI and synthetic biology not only addresses the issue of the lack of standardization in the modernization of traditional Chinese medicine, but also reshapes its scientific paradigm, moving from overall ambiguity to systematic precision, and from master-apprentice inheritance to data-driven (Bülbül et al., 2025). However, several critical challenges must be addressed to realize its full impact, such as data limitation and standardization, model interpretability and scalability of synthetic biology platforms (Hernandez-Boussard et al., 2020; Williams et al., 2023; Jiang et al., 2024).

The path forward hinges on collaborative ecosystems bridging computational science, bioengineering, and TCM epistemology, ensuring innovations like digital twins for herb cultivation and generative AI-designed personalized formulas adhere to both scientific rigor and cultural authenticity.
